# Long-Term Response Without Immune-Related Adverse Events to Atezolizumab Treatment in TMB-High Thymoma: A Case Report from the KOSMOS-II Study

**DOI:** 10.3390/jcm15030958

**Published:** 2026-01-25

**Authors:** In Hee Lee, Moonsik Kim, An Na Seo, Soo Jung Lee, Jee Hyun Kim

**Affiliations:** 1 Department of Oncology/Hematology, School of Medicine, Kyungpook National University, Kyungpook National University Chilgok Hospital, Daegu 41404, Republic of Korea; cakey83@hanmail.net (I.H.L.); majestio@daum.net (S.J.L.); 2 Department of Pathology, School of Medicine, Kyungpook National University, Daegu 41404, Republic of Korea; teiroa83@knu.ac.kr (M.K.); san0729@knu.ac.kr (A.N.S.); 3 Department of Internal Medicine and Genomic Medicine, Seoul National University Bundang Hospital, Seoul National University College of Medicine, Seongnam 13620, Republic of Korea

**Keywords:** thymic epithelial tumor, immune checkpoint inhibitor, tumor mutation burden, atezolizumab

## Abstract

**Background:** Thymic epithelial tumors (TETs), including thymic carcinomas and thymomas, are rare malignancies originating in the mediastinum. Therapeutic options remain limited for patients experiencing disease progression following platinum-based chemotherapy. High tumor mutational burden (TMB) is uncommon in thymic malignancies but may predict response to immunotherapy. We report a patient with TMB-high TET who participated in the KOSMOS-II study in South Korea and achieved a durable response to atezolizumab without developing immune-related adverse events (irAEs). **Case presentation:** A 73-year-old woman who had been treated for thymoma 20 years ago presented with a left neck mass. A biopsy of the neck mass confirmed recurrent thymoma, type B3, and her disease progressed despite platinum-based chemotherapy and subsequent pemetrexed treatment. TMB-high thymoma is very rare, but based on the next-generation sequencing (NGS) results, she was diagnosed with TMB-high (20.3 mutations/Mb) thymoma. As TMB-based immunotherapy is not approved in Korea, she was enrolled in the KOSMOS-II study and initiated on atezolizumab following molecular tumor board review. She achieved stable disease after three cycles and has remained progression-free for 14 months, completing 20 cycles without significant irAEs. Notably, her underlying myasthenia gravis did not worsen during treatment. **Conclusions:** This case demonstrates a favorable outcome with biomarker-directed ICI treatment in recurrent thymoma with limited treatment options, highlighting the importance of appropriate molecular markers to predict drug response. Although TMB-based immunotherapy is FDA-approved in the U.S., it remains unavailable in Korea, underscoring the need to explore flexible access pathways, including the potential use of immunotherapy beyond current indications, to improve treatment options for patients with life-threatening conditions.

## 1. Introduction

Thymic epithelial tumors (TETs), which include thymomas and thymic carcinomas, are rare malignancies in adults but represent the most frequent neoplasms of the anterior mediastinum [1]. Their annual incidence is approximately 0.15 per 100,000. Surgical resection remains the only curative treatment for TETs [2]. However, standard treatment is lacking for patients with recurrent disease [3]. Therapeutic options are particularly limited following the failure of platinum-based chemotherapy, with few prospective studies exploring alternative strategies.

The TMB refers to the total number of somatic non-synonymous mutations in a particular region of the tumor genome. A TMB of ≥10 mutations per megabase was reported to predict a better response to ICIs in several cancers [4]. In thymomas, high TMB is uncommon. The Cancer Genome Atlas (TCGA) Project reported that thymic carcinoma shows a high TMB (21.29 mutations per megabase), whereas thymoma has the lowest average TMB (0.48 mutations per megabase) among adult cancers [5]. High TMB is a predictive marker for response to immune checkpoint inhibitors (ICIs), leading to FDA approval of ICIs for tumors with high TMB [6,7]. However, in TETs, treatment with ICIs is associated with a high incidence of immune-related adverse events (irAEs), requiring careful attention.

Here, we report a case of a patient with TMB-high recurrent thymoma who achieved a durable long-term response to atezolizumab without experiencing severe irAEs. Although, the use of ICIs for TMB-high cancers in not approved in Korea, this patient was able to receive atezolizumab treatment by participating in the KOrean Precision Medicine Networking Group Study of MOlecular profiling-guided therapy based on genomic alterations in advanced Solid tumors (KOSMOS)-II trial [8]. Written informed consent was obtained to publish the patient’s clinical details and images.

## 2. Case

**Figure 1:**
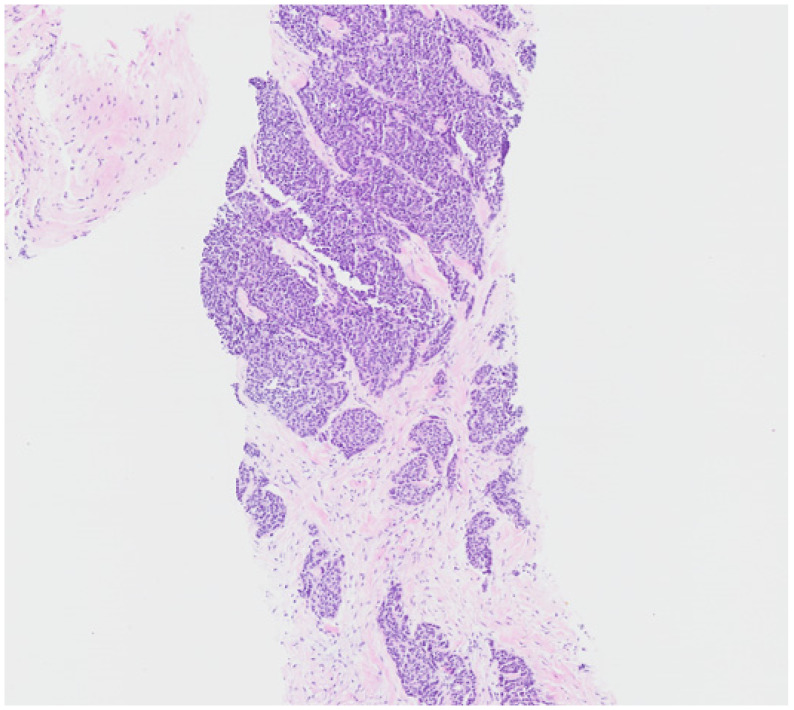
Histopathological features of thymoma tissue. Hematoxylin and eosin (H & E) stain at 50× magnification showing tumor cells infiltrating desmoplastic stroma.

A 73-year-old female patient presented with a left neck mass. Twenty years prior, she had a history of myasthenia gravis, during the investigation of which a thymic mass was discovered and surgically removed. Following thymectomy, she was diagnosed with thymoma and received two cycles of ADOC (doxorubicin, cisplatin, vincristine, and cyclophosphamide) chemotherapy along with radiotherapy. She remained disease-free until the recent presentation with a left neck mass. A core needle biopsy of the left neck mass led to a diagnosis of metastatic thymoma, type B3. Figure 1 shows a representative image of the thymic epithelial tumor. The tumor cells exhibited moderate nuclear atypia with eosinophilic cytoplasm and were found within a fibrotic stroma. PET/CT imaging revealed no distant metastasis. She subsequently underwent six cycles of etoposide and cisplatin (EP) chemotherapy, followed by radiotherapy, achieving a partial response. However, her disease progressed again, and she received eight cycles of pemetrexed. Despite this treatment, the neck mass continued to enlarge, leading to aspiration symptoms during swallowing.

**Figure 2:**
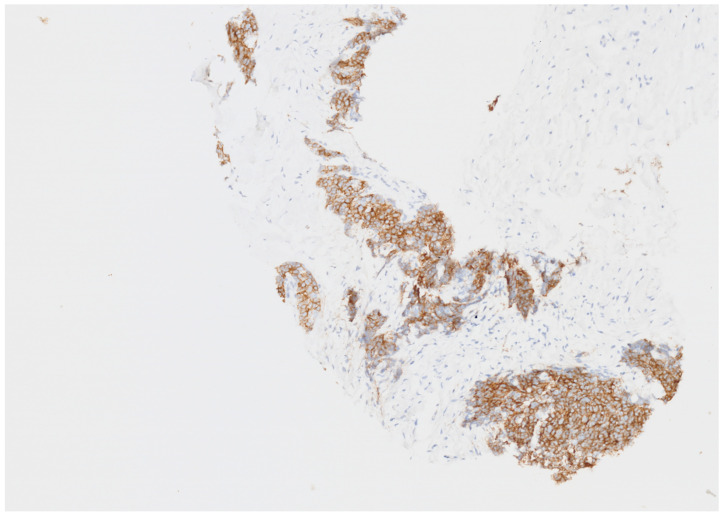
Immunohistochemistry for MLH1. Tumor cells were positive for MLH1 (no loss of expression).

Next-generation sequencing (NGS) testing was performed using biopsy tissue from the neck mass, and a high TMB (20.3 mutations/Mb) was confirmed (Table 1). On DNA sequencing using OncoAccuPanel (hybrid capture-based DNA sequencing using illumina platform), a relatively large number of tier 2 mutations and tier 3 mutations were detected, which suggests a TMB-high type. Estimated TMB was 20.3 muts/Mb. (TMB-high criteria of our institution is more than 20.0 muts/Mb). TMB was calculated as the number of somatic nonsynonymous mutations divided by the targeted coding region (0.65 Mb) and was automatically reported by the OncoAccuPanel analysis pipeline. Although the tumor harbored *MLH1* splice site mutation, it was determined to be microsatellite-stable type on the MSI algorithm of OncoAccuPanel and the absence of *MLH1* nuclear expression on immunostaining (Figure 2). Despite the fact that the tumor harbored a pathogenic splice donor–site mutation, it showed a relatively low proportion of indel mutations and a predominance of missense mutations, which is not consistent with the typical mutational profile of MSI-high tumors. In addition, the MSI algorithm implemented in the OncoAccuPanel classified the tumor as MSS. We further confirmed MSS status by MSI-PCR (Taken together, these findings led us to hypothesize that the MLH1 mutation represents a single (one-hit) event. Pathogenic mutations in *MLH1*, *TP53*, *SMAD2*, *ETV6*, and *DNMT3A* were identified.

High TMB is recognized as a predictive biomarker for response to immunotherapy, and the Food and Drug Administration (FDA) has approved immune checkpoint inhibitors for the treatment of tumors with this characteristic [9]. However, in Korea, immune checkpoint inhibitors are not yet approved for use based on TMB status. This patient was enrolled in the KOSMOS-II study [8], a large-scale nationwide master observational study involving a framework for screening patients with metastatic solid tumors for actionable genetic alterations based on local NGS testing and recommends molecular profiling-guided therapy through a remote and centralized molecular tumor board. Following evaluation by the molecular tumor board (MTB) [10,11], the patient initiated atezolizumab treatment through the therapeutic use of investigational drugs program.

**Figure 3:**
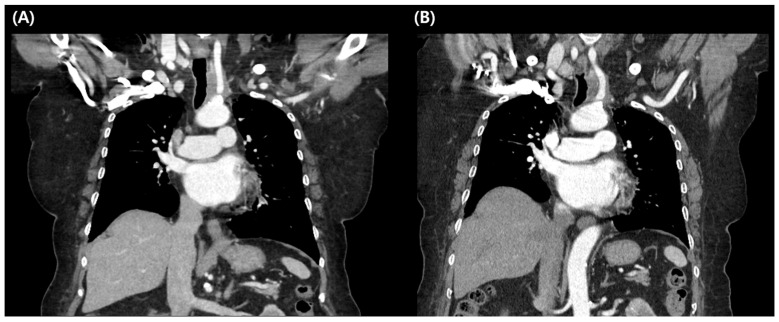
Comparison of serial chest computed tomography (CT) images before and after treatment. (**A**) Pretreatment chest CT; (**B**) chest CT obtained 14 months after atezolizumab treatment. The infiltrative enhancing mass in the left perithyroidal region shows stable disease.

Baseline laboratory tests, including thyroid and adrenal hormone levels, revealed no abnormalities before immunotherapy. The patient commenced her first cycle of atezolizumab and achieved stable disease after three cycles. She has maintained stable disease on atezolizumab and has now completed 20 cycles of treatment and has remained progression-free for 14 months without significant immune-related adverse events (Figure 3). The neck mass in the left perithyroidal region measured 3.3 cm on axial imaging, and after atezolizumab treatment, it decreased to 3.0 cm, consistent with SD.

Regular laboratory tests, including thyroid hormone and ACTH/cortisol levels, were performed, and no significant abnormalities were observed during treatment. Furthermore, during atezolizumab therapy, there was no aspiration or dyspnea, and the patient only complained of mild general weakness. The patient continued maintenance therapy for myasthenia gravis (MG) and regular follow-up with her neurologist throughout the immunotherapy course. She had MGFA class IIA myasthenia gravis and was being treated with pyridostigmine 60 mg four times daily and prednisone 10 mg daily. No exacerbation of MG occurred during treatment, and atezolizumab was well tolerated.

## 3. Discussion

We report a case of recurrent TMB-high thymoma that remained progression-free for more than 14 months with the ICI, atezolizumab, without experiencing severe irAEs. The significance of this case is that TMB-high thymoma is extremely rare, and although the incidence of irAEs during ICIs is high in TETs with MG, this case demonstrated a long-term response without any notable irAEs.

High TMB is a well-established biomarker predictive of response to immunotherapy, particularly immune checkpoint inhibitors [12]. Multiple studies have demonstrated that tumors with high TMB generate more neoantigens, which enhance T-cell recognition and increase the likelihood of a favorable response to immunotherapy [7]. However, in thymoma, the proportion of high-TMB tumors is extremely low, and the overall TMB level is among the lowest across solid cancers [5,13]. In TCGA data, 117 TETs were classified into four molecular subtypes according to genomic hallmarks and their association with survival and WHO histological subtypes. And when compared with 21 other solid cancers, TETs showed the lowest average TMB, with an average of 0.48 mutations per megabase [5].

Consequently, studies on immunotherapy responses in high-TMB thymoma are rare. In high-TMB thymic carcinoma, a few case reports and small-cohort studies have described clinical responses to immune checkpoint inhibitors. Kaneko et al. demonstrated that a patient with MSI-high thymoma and pleural dissemination achieved a partial response after three cycles of pembrolizumab [14]. In the TAPUR basket trial of pembrolizumab for high-TMB solid tumors, efficacy was analyzed separately for colorectal and non-colorectal cohorts. The non-colorectal cohort, which included one case of TET, showed a disease control rate of 45% (one-sided 90% CI, 35–100) and an objective response rate of 26% [15]. This case is unique in that it represents a very rare TMB-high thymoma, and within the TMB-high TETs, the patient achieved a more favorable response than those reported in the previous studies.

According to previous studies that included patients with histologically confirmed TETs, regardless of biomarkers such as TMB, the response to ICIs ranges from 20 to 30%, with a median PFS of 4–6 months [16,17]. In a phase 2 study of 41 patients by Giaccone et al., an ORR of 22.5% (95% CI 10·8–38·5) and a median PFS of 4·2 months (95% CI 2·9–10·3) were reported [16]. Six (15%) patients developed severe autoimmune toxicity in this study. Cho et al. reported a median PFS of 6.1 months and an ORR of 28.6% in thymoma and 19.2% in thymic carcinoma among 33 patients with platinum-resistant TETs treated with pembrolizumab [17]. In this study, five (71.4%) of seven patients with thymoma developed grade ≥ 3 irAEs, including hepatitis, MG, thyroiditis, colitis, and subacute myoclonus.

Furthermore, the patient in this case had underlying MG, and in contrast to previous reports that ICIs can exacerbate pre-existing MG, this case demonstrates successful ICI treatment without any significant MG exacerbation. Mitsune et al. reported that a patient with pre-existing MG in remission developed a relapse of MG symptoms, polymyositis, and rhabdomyolysis after nivolumab treatment [18]. Cooper et al. described a patient with non–small cell lung cancer (NSCLC) and ocular (MG) in virtual remission who developed severe generalized MG, leading to a myasthenic crisis after anti-PD-1 therapy [19]. Plus, in a cohort study of 65 patients, ICI-related MG (new onset or disease flare) occurred in 63 patients (97%), with a median onset of 4 weeks (range, 1–16 weeks) after treatment initiation [20]. In this study, death was reported in 24 patients (38%), with 15 (23%) due to MG complications. Patients who received intravenous immunoglobulin (IVIG) or plasmapheresis (PLEX) as first-line therapy demonstrated superior outcomes compared with those treated with steroids alone. MG is a life-threatening adverse event with acute onset and rapid progression after ICI treatment. This case demonstrates successful ICI treatment without worsening MG through regular laboratory test and neurological follow-up.

TMB has several limitations as a biomarker for immunotherapy response [10]. A standardized method for measuring TMB is lacking, leading to variability in results across sequencing platforms and mutation-calling pipelines. Universal TMB thresholds do not perform consistently across different tumor types [4], and many patients with low TMB respond to immunotherapy, whereas some with high TMB do not respond [10]. In certain cancers, such as GBM, patients with lower-than- median TMB have shown longer survival after ICB treatment compared to those with higher TMB, suggesting that TMB alone has limitations as a predictive marker for immunotherapy response [8]. ICI treatment is not approved for TMB-high tumors in Korea. However, this patient participated in the KOSMOS-II study, and the NGS results were reviewed by the MTB, which included medical oncologists, pathologists, and a bioinformatics specialist (BI). Based on the MTB’s recommendation, atezolizumab was offered as an appropriate treatment for the TMB-high tumor. The patient remains on immunotherapy with no severe treatment-related adverse events observed to date. However, given the high risk of irAEs, careful risk–benefit assessment and shared decision-making are required.

This case demonstrates a favorable outcome with biomarker-directed ICI treatment in recurrent TET with limited treatment options, highlighting the importance of appropriate markers to predict drug response. Although TMB-based immunotherapy is FDA-approved in the U.S., it remains unavailable in Korea. Its limited availability in Korea underscores the need to explore flexible access pathways, including the potential use of immunotherapy beyond current indications, to improve treatment options for patients with life-threatening conditions.

## Figures and Tables

**Table 1:** NGS report.

Gene	HGVS Coding	HGVS Protein	Consequence	Variant Allele Frequency (%)	Clinical Significance
*SMAD2*	c.544C>T	p.R182Ter	Nonsense mutation	29.01	Likely pathogenic
*MLH1*	c.1667_1667+2del		Splice donor site mutation	38.06	Likely pathogenic
*TP53*	c.524G>A	p.R175H	Missense mutation	16.43	Likely pathogenic
*ETV6*	c.1105C>T	p.R369W	Missense mutation	28.26	Likely pathogenic
*DNMT3A*	c.891_910delinsC	p.W297CfsTer13	Frameshift mutation	3.05	Likely pathogenic
*ARID1B*	c.3619C>A	p.P1207T	Missense mutation	30.94	Variants of uncertain significance
*BRAF*	c.914C>A	p.A305E	Missense mutation	23.82	Variants of uncertain significance
*DDR1*	c.2423G>A	p.R808H	Missense mutation	26.54	Variants of uncertain significance
*DDR2*	c.994C>T	p.R332W	Missense mutation	13.53	Variants of uncertain significance
*FLT1*	c.245G>A	p.G82E	Missense mutation	29.89	Variants of uncertain significance
*HDAC9*	c.2636G>A	p.G879D	Missense mutation	20.64	Variants of uncertain significance
*MET*	c.306C>G	p.S102R	Missense mutation	34.95	Variants of uncertain significance
*PBRM1*	c.3760C>T	p.L1254F	Missense mutation	27.68	Variants of uncertain significance

DNA sequencing using the OncoAccuPanel (a hybrid capture-based DNA sequencing assay on the Illumina platform) identified a relatively high number of tier 2 and tier 3 mutations, suggesting a TMB-high tumor. Estimated TMB was 20.3 muts/Mb.

## Data Availability

The original contributions presented in this study are included in the article. Further inquiries can be directed to the corresponding author.
